# Attribute-based cross-classification reveals sex- and age-specific prognostic impact of anemia in ADPKD

**DOI:** 10.1007/s10157-026-02867-0

**Published:** 2026-04-29

**Authors:** Kosaku Nitta, Hiroshi Kataoka, Yusuke Ushio, Shun Manabe, Shiho Makabe, Shigeru Otsubo, Norio Hanafusa, Ken Tsuchiya, Junichi Hoshino, Toshio Mochizuki

**Affiliations:** 1 Department of Nephrology, Tokyo Women’s Medical University, 8-1 Kawada-Cho, Shinjuku-Ku, Tokyo, 162-8666 Japan; 2 Department of Clinical Engineering, Faculty of Human Care at Makuhari, Tohto University, Chiba, Japan; 3 Department of Blood Purification, Tokyo Women’s Medical University, Tokyo, Japan; 4 PKD Nephrology Clinic, Tokyo, Japan

**Keywords:** Anemia, Renal prognosis, Attribute-based medicine, Cross-classification, Sex differences

## Abstract

**Background:**

Anemia is less prevalent in autosomal dominant polycystic kidney disease (ADPKD) owing to preserved erythropoietin production. However, its impact on kidney prognosis remains unclear. Given sex-related differences in hemoglobin (Hb) levels, we hypothesized that the prognostic relevance of anemia may vary by sex and age. Therefore, we aimed to identify subgroup-specific risk patterns using an attribute-based cross-classification approach to support individualized anemia management in ADPKD.

**Methods:**

We analyzed 552 Japanese patients with ADPKD from a single-center cohort. The primary outcome was a ≥ 30% decline in the estimated glomerular filtration rate (eGFR) or initiation of renal replacement therapy. Cox regression analysis was used to assess the association between Hb and kidney outcomes. Subgroup analyses were performed using cross-classification by sex and age (< 50 or ≥ 50 years). Anemia was defined using multiple Hb thresholds (< 11, < 12, and < 13 g/dL).

**Results:**

Lower Hb levels were independently associated with worse renal outcomes (hazard ratio [HR] per 1 g/dL increase: 0.83). Cross-classified analyses revealed distinct risk patterns. Anemia (Hb level < 13.0 g/dL) significantly increased the risk in young (HR: 2.92) and old men (HR: 3.84). In women, anemia defined as a Hb level < 12.0 g/dL was associated with adverse outcomes in both age groups (HR: 1.98 in < 50 years; HR: 2.08 in ≥ 50 years).

**Conclusion:**

Anemia is a significant prognostic marker for kidney disease progression in ADPKD. Its prognostic impact differs by sex and age, suggesting the need for attribute-based, individualized hemoglobin thresholds rather than uniform cutoffs, to optimize risk stratification and clinical assessment.

**Supplementary Information:**

The online version contains supplementary material available at 10.1007/s10157-026-02867-0.

## Introduction

Autosomal dominant polycystic kidney disease (ADPKD) is a hereditary condition marked by progressive cyst formation and renal function decline. Established risk factors for poor outcomes include *PKD1* mutations, hypertension, proteinuria, and increased total kidney volume (TKV) [1–4]. Anemia is a known predictor of chronic kidney disease (CKD) progression [5–7]; however, it is less common in ADPKD owing to erythropoietin (EPO) production by renal cysts [8–10]. Its prognostic value in ADPKD remains unclear [2, 11, 12].

Recent advances in personalized and attribute-based medicine have shown the importance of tailoring CKD management to individual patient characteristics [13–21]. The 2024 Kidney Disease: Improving Global Outcomes (KDIGO) conference reinforced this need, emphasizing personalized care over uniform treatment models [22]. Sex, age, and disease stage significantly influence disease progression and therapeutic response [13, 15, 17, 22–24].

Conventional subgroup analyses typically examine single attributes, such as sex or age, which may overlook interactions between overlapping factors [25]. In contrast, cross-classification analyses consider two attributes together, revealing risk profiles not seen in simpler models, and are commonly used in marketing research and have been increasingly applied in attribute-based medicine [18, 19, 21]. They classify patients into four subgroups that capture direct and indirect effects [19] and clarify which attributes most influence prognosis [21] (Supplementary Fig. 1). For kidney disease, physiological differences between younger men and older women support tailored risk assessment [18, 26], as the impact of anemia may vary across these groups [21].

Sex-based differences in CKD and anemia are well established [11, 21, 27–30]. The 2020 KDIGO conference emphasized individualized anemia management based on sex, age, and comorbidities [31], although current standards remain sex-neutral due to limited evidence.

Given the progressive nature and heterogeneity of ADPKD in clinical presentation, understanding how anemia and other prognostic factors vary across patient subgroups is essential. In this study, we analyzed data from a large single-center Japanese cohort with ADPKD using a sex and age-based cross-classification model to assess the prognostic impact of anemia and explore its potential for attribute-based risk stratification.

## Materials and methods

### Study design and population

The records of 586 outpatients with ADPKD who visited the Kidney Center at Tokyo Women’s Medical University Hospital (Tokyo, Japan) between July 2003 and July 2019 were reviewed. In accordance with routine clinical practice in Japan, patients who were already undergoing dialysis therapy were excluded (n = 33). One patient younger than 15 years who was managed in a pediatric setting was also excluded, resulting in an analytic cohort representative of patients routinely followed in adult nephrology practice.

Age 50 years was selected a priori as a clinically meaningful cut-off reflecting major life-stage transitions, including menopause in women and age-related hormonal and metabolic changes in men, which may influence anemia status and disease progression. This resulted in a final analytic cohort of 552 patients (Supplementary Fig. 2). ADPKD was diagnosed using the previously described criteria [32]. Clinical data were collected from baseline examinations, and the patients were prospectively observed to evaluate outcomes. The Research Ethics Committee of Tokyo Women’s Medical University (No. 5118) approved this study under the 1964 Declaration of Helsinki and its later amendments or equivalent ethical standards. Passive informed consent (opt-out) was obtained from patients, and all data were anonymously analyzed. Additional methodological details are provided in the supplementary material (Supplementary Methods). Comorbidities, including hypertension, diabetes mellitus, and dyslipidemia, were defined based on standard clinical criteria (see Supplementary Methods for detailed definitions). Anemia was classified into three categories based on hemoglobin (Hb) levels: < 11 g/dL, < 12 g/dL, and < 13 g/dL, with anemia defined by Hb levels in these ranges or by the use of iron or erythropoiesis-stimulating agents (ESAs). Categorical anemia definitions were evaluated in separate multivariable models replacing continuous hemoglobin and were treated as prespecified sensitivity analyses to assess the robustness and clinical interpretability of the findings.

Patients administering tolvaptan were excluded from the renal prognosis analysis, and those who initiated tolvaptan therapy during follow-up were censored. Deaths during follow-up were treated as censoring events in all time-to-event analyses, given the limited number of deaths observed. Formal competing risk regression using the Fine–Gray model was therefore not performed. To evaluate the robustness and generalizability of the findings, a series of prespecified sensitivity analyses were conducted, including restriction to adult patients, exclusion of patients treated with tolvaptan at baseline, and alternative adjustments for kidney volume using height-adjusted TKV and Mayo imaging classification (details in Supplementary Tables 1–5). The participants were followed up until June 30, 2024.

### Outcome evaluations

Renal outcome was defined as a ≥ 30% reduction in eGFR or the initiation of renal replacement therapy.

### Statistical analyses

Continuous variables are expressed as means ± standard deviations or medians (ranges), as appropriate, whereas discrete variables are expressed as percentages. For continuous variables, the Mann–Whitney U and unpaired t-tests were conducted according to data distribution, after assessing normality. The chi-square or Fisher’s exact test was used to analyze categorical variables. Multivariable Cox regression analyses were conducted to calculate hazard ratios (HRs) and 95% confidence intervals (CIs) for the primary endpoint. Variables of interest and established risk factors for renal outcomes based on existing knowledge were included in the multivariable models [14, 18, 33, 34]. Standard methods were used to estimate the sample size for multivariable Cox regression analyses, with at least five outcome events required per independent variable [35, 36]. Statistical significance was set at a two-sided P value < 0.05, and all analyses were conducted using JMP Pro software (version 18.0.0; SAS Institute, Cary, NC, USA).

## Results

### Patient characteristics

Table 1 summarizes the baseline characteristics of 552 patients with ADPKD. The median age was 43 years, mean eGFR was 55.9 mL/min/1.73 m^2^, and mean TKV was 1335.4 mL. Renal function declined with age, especially in men; older men had the lowest eGFR, whereas younger women had the highest. Hb levels were higher in men than in women and declined with age. Baseline use of erythropoiesis-stimulating agents, iron supplementation, and tolvaptan was uncommon and is summarized in Table 1. Younger men had the highest mean Hb (13.9 g/dL), whereas older women had the lowest (11.5 g/dL). Anemia (Hb level < 12.0 g/dL) was present in 32.6% of patients, with prevalence highest in older women (52.3%) and lowest in younger men (17.2%), based on sex and age-based cross-classification. Metabolic comorbidities, such as hypertension (52.6%), hyperuricemia, and dyslipidemia, were more common in older patients, particularly men, than in younger patients.

**Table 1:** Characteristics of the entire cohort and sub-cohorts cross-classified by sex and age

Variables	Entire cohort n = 552	Men, < 50 years n = 168	Men, ≥ 50 years n = 75	Women, < 50 years n = 202	Women, ≥ 50 years n = 107	P–value
Clinical Findings						
Age (years)	43 (15–86) [552]	39 (15–49)	59 (50–86)	36 (15–49)	59 (50–81)	< 0.001*
Systolic blood pressure (mmHg)	127.4 ± 13.4 [513]	129.2 ± 12.5	131.7 ± 11.9	121.6 ± 13.3	131.9 ± 12.6	< 0.001*
Diastolic blood pressure (mmHg)	79.7 ± 9.8 [510]	82.6 ± 10.1	79.3 ± 8.2	77.3 ± 10.1	79.7 ± 8.3	< 0.001*
Body mass index (kg/m^2^)	22.3 ± 3.3 [525]	23.8 ± 3.6	23.1 ± 2.3	21.1 ± 2.9	21.6 ± 3.1	< 0.001*
Laboratory Findings						
Hemoglobin (g/dL)	12.7 ± 1.9 [540]	13.9 ± 1.9	12.7 ± 1.7	12.3 ± 1.6	11.5 ± 1.7	< 0.001*
Anemia of Hb < 11.0 g/dL, n (%)	117 (21.1) [552]	23 (13.6)	16 (21.3)	42 (20.8)	36 (33.6)	0.001*
Anemia of Hb < 12.0 g/dL, n (%)	180 (32.6) [552]	29 (17.2)	26 (34.7)	69 (34.2)	56 (52.3)	< 0.001*
Anemia of Hb < 13.0 g/dL, n (%)	283 (51.3) [552]	39 (23.1)	38 (50.7)	124 (61.4)	82 (76.6)	< 0.001*
Serum creatinine (mg/dL)	1.65 ± 1.65 [552]	1.90 ± 1.74	2.29 ± 1.36	1.00 ± 1.15	2.06 ± 2.04	< 0.001*
eGFR (mL/min/1.73m^2^)	55.7 ± 30.4 [552]	54.5 ± 27.9	32.9 ± 17.2	75.3 ± 27.3	36.8 ± 22.3	< 0.001*
Uric acid (mg/dL)	5.7 ± 1.7 [526]	6.6 ± 1.3	6.7 ± 1.5	4.4 ± 1.3	5.8 ± 1.3	< 0.001*
U-Prot (grade 0–3)	0 (0–3) [551]	0 (0–3)	0.5 (0–2)	0 (0–2)	0.5 (0–3)	< 0.001*
TKV (mL)	1337.8 ± 1006.9 [473]	1755.9 ± 1287.4	1634.9 ± 916.9	919.2 ± 612.3	1249.8 ± 783.1	< 0.001*
htTKV (mL/m)	809.8 ± 591.5 [459]	1014.3 ± 745.4	966.8 ± 537.5	580.6 ± 389.5	796.3 ± 489.5	< 0.001*
Mayo imaging classification 1A/Class1B/Class1C/Class1D/Class1E/Class2, n (%)	52 (11.5)/149 (32.9)/135 (29.8)/81 (17.9)/36 (7.9)/0 (0.0) [453]	8 (5.4)/25 (17.0)/42 (28.6)/43 (29.3)/29 (19.7)/0 (0.0)	8 (13.6)/25 (42.4)/20 (33.9)/6 (10.2)/0 (0.0)/0 (0.0)	23 (13.7)/59 (35.1)/49 (29.2)/30 (17.6)/7 (4.2)/0 (0.0)	13 (16.5)/40 (50.6)/24 (30.4)/2 (2.5)/0 (0.0)/0 (0.0)	< 0.001*
Mayo imaging classification Class1C–1E, n (%)	252 (55.6) [453]	114 (77.6)	26 (44.1)	86 (51.9)	26 (32.9)	< 0.001*
Concomitant drugs						
Antihypertensive agents, n (%)	244 (44.2) [552]	93 (55.4)	52 (69.3)	36 (17.8)	63 (58.9)	< 0.001*
Antidyslipidemic agents, n (%)	35 (6.3) [552]	9 (5.4)	13 (17.3)	3 (1.5)	10 (9.4)	< 0.001*
Erythropoiesis-stimulating agents, n (%)	45 (8.2) [552]	9 (5.4)	8 (10.7)	15 (7.4)	13 (12.2)	0.186
Iron supplementation, n (%)	21 (3.8) [552]	2 (1.2)	0 (0.0)	13 (6.4)	6 (5.6)	0.012*
Tolvaptan, n (%)	7 (1.3) [552]	5 (3.0)	1 (1.3)	1 (0.5)	0 (0.0)	0.010*
Comorbidities						
Hypertension, n (%)	291 (52.7) [552]	109 (64.9)	60 (80.0)	54 (26.7)	68 (63.6)	< 0.001*
Hyperuricemia, n (%)	165 (29.9) [552]	78 (46.4)	49 (65.3)	11 (5.5)	27 (25.2)	< 0.001*
Hypertriglyceridemia, n (%)	106 (19.2) [552]	48 (28.6)	25 (33.3)	12 (5.9)	21 (19.6)	< 0.001*
Low HDL cholesterol, n (%)	75 (13.6) [552]	26 (15.5)	17 (22.7)	14 (7.0)	18 (16.8)	< 0.001*
High LDL cholesterol, n (%)	74 (13.4) [552]	23 (13.7)	14 (18.7)	11 (5.5)	26 (24.3)	< 0.001*
Diabetes, n (%)	13 (2.4) [552]	4 (2.4)	4 (5.3)	1 (0.5)	4 (3.7)	0.010*

*P < 0.05. Continuous values are expressed as the mean ± standard deviation or median (minimum–maximum). Discrete variables are expressed as n (%). Values for number of patients are shown in []

Anemia of Hb < 11.0 g/dL, Hb level < 11 g/dL, or taking iron or erythropoiesis-stimulating agents (ESA); Anemia of Hb < 12.0 g/dL, Hb level < 12 g/dL, or taking iron or ESA; Anemia of Hb < 13.0 g/dL, Hb level < 13 g/dL, or taking iron or ESA

*n* number, *%* percentage, *eGFR* estimated glomerular filtration rate, *U*-*Prot* Urinary protein excretion, *CKD* Chronic kidney disease, *TKV* Total kidney volume, *htTKV* Height-adjusted total kidney volume, *HDL* High-density lipoprotein, *LDL* Low-density lipoprotein

### High-priority attributes for renal function deterioration in age- and sex-based cross-classification

**Fig. 1:**
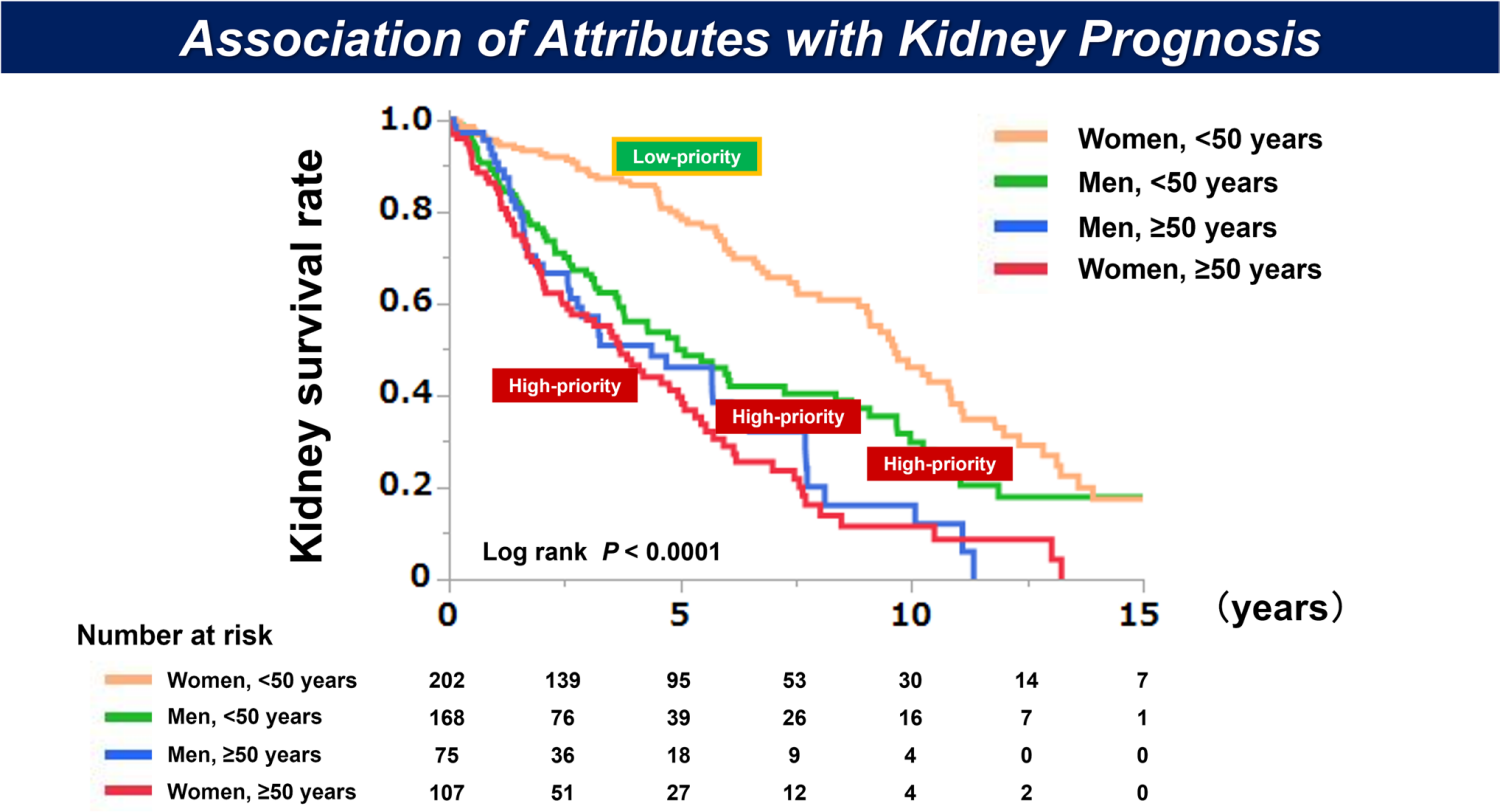
Kidney prognosis stratified by sex and age in patients with ADPKD, Kaplan–Meier curves illustrate renal survival, defined as time to a ≥ 30% decline in the eGFR or initiation of renal replacement therapy, stratified by sex (men vs. women) and age (< 50 vs. ≥ 50 years). Among the four subgroups, younger women (< 50 years) have the most favorable prognosis, whereas younger men (< 50 years) show the poorest renal outcomes. The difference across groups is statistically significant (log-rank P < 0.001). These findings highlight the importance of attribute-based stratification in predicting renal outcomes in ADPKD. *ADPKD* autosomal dominant polycystic kidney disease, *vs*. versus, *eGFR* estimated glomerular filtration rate

Kaplan–Meier analysis based on sex- and age-based cross-classification revealed significant differences in kidney prognosis among the four subgroups (log-rank P < 0.0001). Notably, younger women had the most favorable renal outcomes, whereas younger men exhibited the poorest. These distinct trajectories emphasize the prognostic value of attribute-based cross-classification in ADPKD (Fig. 1).

### Renal prognostic indicators in patients with ADPKD (entire cohort)

The median follow-up was 5.3 years, during which 266 patients reached the primary kidney outcome. During follow-up, 17 patients died before reaching the primary kidney outcome and were censored at the time of death. Multivariable Cox regression analysis identified age, eGFR, urinary protein excretion (U-Prot), TKV, Hb, and hypertension as significant prognostic factors (Table 2). In the primary multivariable model, hemoglobin analyzed as a continuous variable was independently associated with renal outcomes (HR per 1 g/dL increase: 0.83; P < 0.001).

**Table 2:** Multivariable cox regression analyses for kidney outcomes in the analytic cohort

Variables	Hazard Ratio (95% CI)	P–value
Age (10 year increase)	0.80 (0.70–0.91)	0.001*
Woman (vs. man)	1.05 (0.72–1.55)	0.798
eGFR (10-mL/min/1.73m^2^ increase)	0.64 (0.57–0.71)	< 0.001*
U-Prot (grade 0–3)	1.87 (1.49–2.32)	< 0.001*
TKV (100 mL/m increase)	1.02 (1.01–1.03)	0.001*
Hypertension (vs. no)	1.57 (1.14–2.16)	0.006*
Uric acid (1 mg/dL increase)	1.01 (0.89–1.15)	0.867
Hb (1 g/dL increase): primary exposure	0.83 (0.75–0.91)	< 0.001*
Categorical analyses (alternative hemoglobin definitions; separate models)		
Anemia of Hb < 11.0 g/dL	[1.38 (0.94–2.00)]	[0.093]
Anemia of Hb < 12.0 g/dL	[1.69 (1.19–2.40)]	[0.003*]
Anemia of Hb < 13.0 g/dL	[1.60 (1.11–2.31)]	[0.012*]
Anemia of Hb < 12.0 g/dL in men, Hb < 11.0 g/dL in women	[1.51 (1.04–2.17)]	[0.029*]
Anemia of Hb < 13.0 g/dL in men, Hb < 12.0 g/dL in women	[1.81 (1.28–2.56)]	[< 0.001*]

*P < 0.05. Analyses were performed in the analytic cohort with complete data for all covariates. The primary multivariable model analyzed hemoglobin as a continuous variable (follow-up duration = 5.3 years; n = 443, events = 219). Categorical anemia definitions were evaluated in separate multivariable models replacing continuous hemoglobin and are presented as secondary analyses. Variables representing established risk factors for kidney disease progression in autosomal dominant polycystic kidney disease and chronic kidney disease were included in the multivariable models. Hazard ratios shown in brackets [] represent results of prespecified sensitivity analyses using alternative hemoglobin thresholds (< 11.0, < 12.0, and < 13.0 g/dL; < 12.0 g/dL in men and < 11.0 g/dL in women; and < 13.0 g/dL in men and < 12.0 g/dL in women) and were not included in the primary model. Anemia was defined by hemoglobin thresholds or the use of iron supplementation or erythropoiesis-stimulating agents

*CI* Confidence interval, *vs*. versus, *eGFR* estimated glomerular filtration rate, *Hb* hemoglobin, *TKV* total kidney volume, *U*-*Prot* Urinary protein excretion

**Fig. 2:**
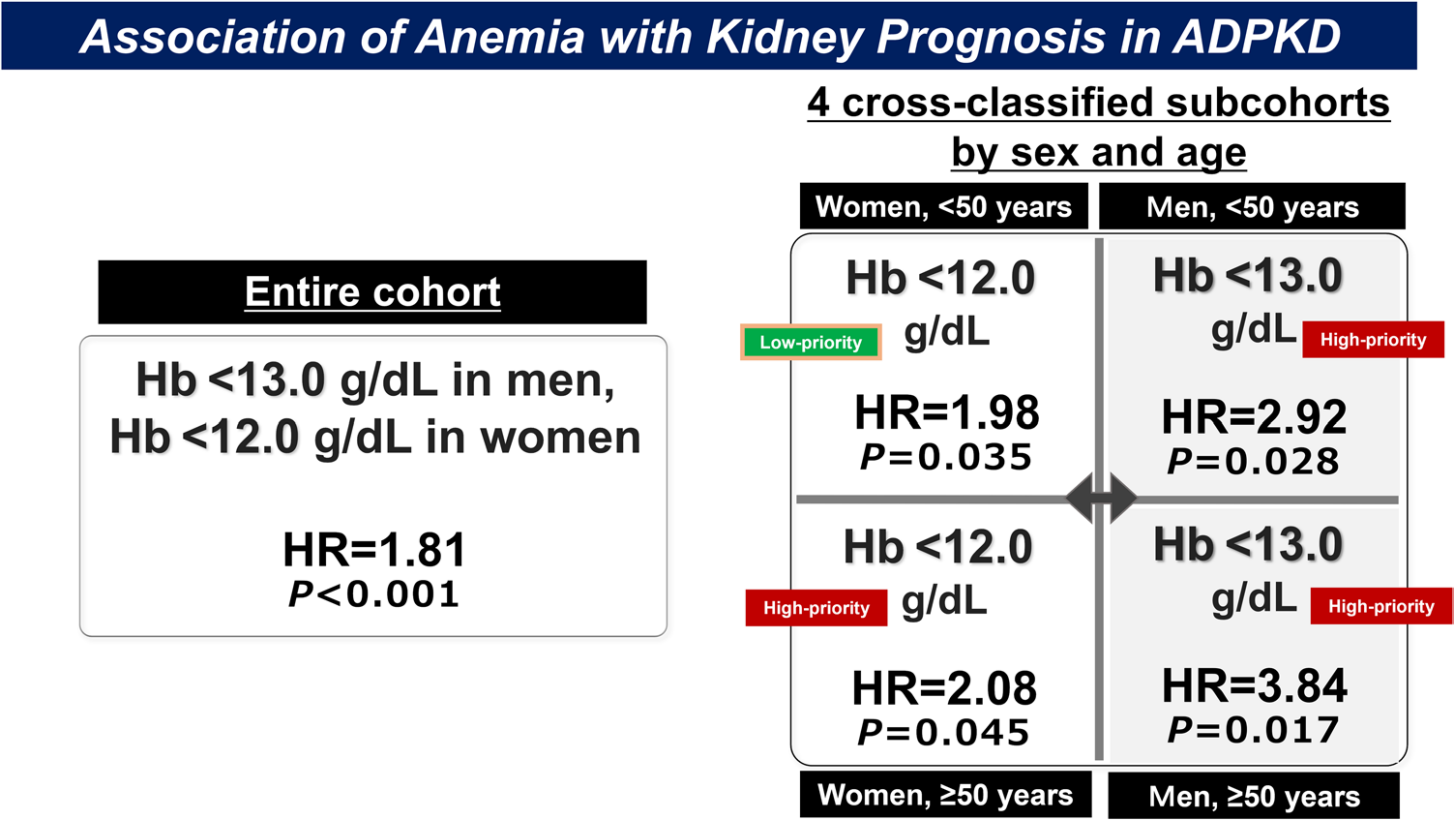
Cox regression-based hazard ratios (HRs) of anemia for renal outcomes in ADPKD, The left panel, based on Table 2, shows HRs in the entire cohort. The right panel, based on Table 3, presents corresponding HRs in four subgroups defined by cross-classification by sex and age (< 50 or ≥ 50 years). This figure underscores the prognostic value of individualized assessment based on patient attributes. *ADPKD* autosomal dominant polycystic kidney disease; Hb, hemoglobin

Sensitivity analyses using various Hb thresholds confirmed this association. Anemia defined as Hb levels < 12.0 g/dL (HR: 1.69; P = 0.003) and < 13.0 g/dL (HR: 1.60; P = 0.012) significantly predicted poor outcomes, whereas Hb levels < 11.0 g/dL did not. Sex-specific thresholds also showed prognostic value (Fig. 2):

- Hb levels < 12.0 g/dL in men and < 11.0 g/dL in women (HR: 1.51; P = 0.029)
- Hb levels < 13.0 g/dL in men and < 12.0 g/dL in women (HR: 1.81; P < 0.001)

Across all sensitivity analyses, including alternative hemoglobin thresholds, restriction to patients aged ≥ 18 years, exclusion of baseline tolvaptan users, and adjustment using height-adjusted TKV or Mayo imaging classification, the associations between lower hemoglobin levels and adverse kidney outcomes remained consistent in both direction and magnitude (Supplementary Tables 1–4). Additional sensitivity analyses incorporating metabolic comorbidities (body mass index, diabetes, and lipid abnormalities) yielded consistent results (Supplementary Table 5).

To further illustrate the prognostic impact of anemia, Kaplan–Meier curves were generated using the hemoglobin cutoff values that yielded the highest hazard ratios in the corresponding multivariable Cox models. These curves are shown for the entire analytic cohort and for subgroups cross-classified by sex and age (< 50 and ≥ 50 years) in Supplementary Fig. 3.

### Renal prognostic indicators in patients with ADPKD (attribute-based cross-classification by sex and age)

Attribute-based cross-classification by sex and age revealed distinct prognostic patterns (Table 3). In younger men (< 50 years), lower eGFR, larger TKV, hypertension, and Hb level < 13.0 g/dL (HR: 2.92) were significantly associated with poor outcomes. Higher Hb levels were protective (HR per 1 g/dL increase: 0.78). In older men (≥ 50 years), U-Prot and Hb level < 13.0 g/dL (HR: 3.84) were the primary predictors of adverse outcomes.

**Table 3:** Multivariable Cox regression analyses for kidney outcomes in attribute-based cross-classified sub-cohorts by sex and age (analytic cohort)

	Men, < 50 years, follow-up = 5.3 years, n = 146, event = 67		Men, ≥ 50 years, follow-up = 3.9 years, n = 61, event = 36		Women, < 50 years, follow-up = 6.2 years, n = 165, event = 65		Women, ≥ 50 years, follow-up = 4.8 years, n = 82, event = 56	
Variables	Hazard ratio (95% CI)	P–value	Hazard ratio (95% CI)	P–value	Hazard ratio (95% CI)	P–value	Hazard ratio (95% CI)	P–value
Age (10-year increase)	0.68 (0.43–1.08)	0.097	0.78 (0.51–1.16)	0.228	1.00 (0.64–1.59)	0.996	0.73 (0.48–1.10)	0.134
eGFR (10-mL/min/1.73m^2^ increase)	0.59 (0.46–0.75)	< 0.001*	0.69 (0.44–1.01)	0.076	0.74 (0.60–0.91)	0.004*	0.61 (0.48–0.77)	< 0.001*
U-Prot (grade 0–3)	1.41 (0.96–2.04)	0.072	2.64 (1.43–4.81)	0.002*	1.35 (0.75–2.36)	0.306	3.55 (2.24–5.60)	< 0.001*
TKV (100 mL/m increase)	1.02 (1.00–1.04)	0.014*	0.98 (0.94–1.02)	0.333	1.10 (1.05–1.15)	< 0.001*	0.99 (0.95–1.03)	0.644
Hypertension (vs. no)	2.48 (1.30–5.05)	0.008*	0.52 (0.22–1.32)	0.149	1.04 (0.55–1.90)	0.907	1.50 (0.79–2.91)	0.220
Uric acid (1 mg/dL increase)	1.03 (0.85–1.24)	0.751	0.88 (0.65–1.20)	0.396	0.95 (0.70–1.30)	0.753	1.23 (0.91–1.67)	0.187
Hb (1 g/dL increase)	[0.78 (0.63–0.95)]	[0.015*]	[0.77 (0.54–1.12)]	[0.163]	[0.86 (0.69–1.06)]	[0.150]	[0.69 (0.52–0.91)]	[0.008*]
Anemia of Hb < 11.0 g/dL	[1.83 (0.69–4.78)]	[0.222]	[1.24 (0.40–3.52)]	[0.696]	[1.16 (0.55–2.43)]	[0.703]	[2.17 (0.90–5.09)]	[0.078]
Anemia of Hb < 12.0 g/dL	[1.65 (0.60–4.68)]	[0.336]	[2.45 (0.93–6.57)]	[0.069]	1.98 (1.04–3.68): primary exposure	0.035*	2.08 (1.01–4.29): primary exposure	0.045*
Anemia of Hb < 13.0 g/dL	2.92 (1.13–7.61): primary exposure	0.028*	3.84 (1.29–12.03): primary exposure	0.017*	[1.15 (0.62–2.18)]	[0.657]	[0.98 (0.40–2.54)]	[0.969]

^*^P < 0.05. Analyses were conducted in patients aged ≥ 15 years. All analyses were performed in the analytic cohort with complete data for all covariates. For hemoglobin, three prespecified, clinically relevant thresholds (< 11.0, < 12.0, and < 13.0 g/dL) were evaluated in each sex- and age-stratified subgroup. The multivariable analyses present the results for the threshold associated with the highest hazard ratio within this prespecified range, which is shown as the primary result. Hemoglobin analyzed as a continuous variable and the remaining thresholds are shown in brackets [] as sensitivity analyses. Variables representing established risk factors for kidney disease progression in autosomal dominant polycystic kidney disease and chronic kidney disease were included in all models. Anemia was defined by hemoglobin thresholds and/or the use of iron supplementation or erythropoiesis-stimulating agents

*CI* Confidence interval, *eGFR* estimated glomerular filtration rate, *Hb* hemoglobin, *U*-*Prot* Urinary protein excretion, *vs*. versus

Among younger women (< 50 years), lower eGFR, greater TKV, and Hb level < 12.0 g/dL (HR: 1.98) were associated with worse prognosis. In older women (≥ 50 years), U-Prot, lower eGFR, and Hb level < 12.0 g/dL (HR: 2.08) were adverse factors. Higher Hb levels remained protective (HR: 0.69).

These findings underscore the clinical utility of attribute-based cross-classification in identifying subgroup-specific risk profiles in ADPKD (Fig. 2). To further illustrate the prognostic impact of anemia within this framework, Kaplan–Meier curves stratified by sex and age were additionally generated using the hemoglobin cutoff values that yielded the highest hazard ratios in the corresponding multivariable Cox models. These curves, shown for the entire cohort and for cross-classified subgroups, are presented in Supplementary Fig. 3. These cross-classified analyses were prespecified, and sensitivity analyses restricted to patients aged ≥ 18 years were also performed to ensure the robustness of subgroup-specific associations (Supplementary Table 6).

## Discussion

In this large single-center cohort of Japanese patients with ADPKD, we evaluated multiple Hb thresholds for predicting kidney outcomes. We identified general (Hb level < 12 g/dL) and sex-specific cutoffs (< 13 g/dL in men, < 12 g/dL in women), suggesting that anemia may represent a clinically relevant prognostic marker rather than a uniform consequence of kidney dysfunction. Given the observational design of this study, our findings should not be interpreted as evidence of a causal relationship between anemia and renal outcomes, but rather as indicating that hemoglobin levels serve as a clinically relevant risk stratification marker in ADPKD.

Although anemia is less common in ADPKD due to cyst-driven erythropoietin (EPO) production [8–10], anemia has been associated with rapid eGFR decline and progression to end-stage kidney disease in CKD [5, 7]. Previous studies in ADPKD were limited by small sample sizes [11, 12]; however, our larger cohort confirms that lower Hb levels are significantly associated with faster renal function decline and increased risk of renal replacement therapy. Importantly, regardless of causality, hemoglobin levels consistently demonstrated independent prognostic value across multiple models and sensitivity analyses, supporting their robustness as a clinically meaningful prognostic marker. These associations are pathophysiologically plausible: in early ADPKD, hypoxia induces HIF-mediated EPO synthesis [37], whereas progressive fibrosis and ischemia may eventually impair EPO production [38]. In addition, activation of hypoxia-inducible pathways, including HIF-1α, has been implicated in cyst growth and disease progression [39–41], suggesting that anemia may both reflect and exacerbate underlying renal injury.

A key strength of our study is the use of attribute-based cross-classification, which enabled identification of subgroup-specific risk profiles that may be obscured by conventional single-attribute analyses [18–20]. Rather than attempting to reduce heterogeneity, this approach is specifically designed to capture and characterize clinically meaningful heterogeneity inherent in real-world ADPKD populations. We observed distinct, attribute-dependent risk patterns: anemia was more prevalent and prognostically relevant in older women, whereas the combination of anemia, lower eGFR, and larger TKV identified a particularly high-risk phenotype in younger men. Importantly, while these distributions may partly reflect subgroup imbalance, they are unlikely to be explained solely by selection bias and likely represent intrinsic disease heterogeneity in pre-dialysis ADPKD, where rapidly progressive phenotypes are more frequently encountered in routine clinical practice. These findings indicate that the prognostic impact of anemia is not uniform but context-dependent, being modified by interactions between sex and age. Such patterns provide mechanistic support for sex-specific hemoglobin thresholds [11] and reinforce the clinical utility of an attribute-based medicine framework [18–20].

Sex-specific differences in the prognostic impact of anemia may reflect underlying physiological mechanisms. Men typically have higher baseline hemoglobin levels but may be more susceptible to anemia-related hypoxia due to androgen effects and higher metabolic demands [18, 42, 43], which may partly explain the stronger association between anemia and adverse kidney outcomes observed in men. Consistent with this concept, Kataoka et al. [21] demonstrated that renal outcomes in patients with chronic kidney disease and anemia are differentially influenced by hemoglobin levels and erythropoiesis-stimulating agent (ESA) responsiveness according to sex and age (BRIGHTEN study).

In contrast to prior models such as the PRO-PKD score, in which male sex has been identified as a risk factor [33], sex was not independently associated with renal outcomes in our cohort after adjustment for established disease severity markers. This discrepancy may reflect differences in cohort characteristics and analytical frameworks, particularly the inclusion of a real-world pre-dialysis population and explicit adjustment for kidney function, proteinuria, and kidney volume. In this context, cross-classification may redistribute variance across interacting attributes rather than capturing main effects alone. Thus, rather than a simple main effect of sex, our findings support a context-dependent role of sex within an attribute-based framework [11, 21, 27–30].

Although the present study did not evaluate anemia treatment effects, it delineated age- and sex-specific anemia risk profiles in patients with ADPKD using an attribute-based cross-classification approach. As ADPKD is a genetically determined disease, it is not primarily characterized by sex-specific pathogenesis, and observed sex-related differences in prognosis may reflect differences in disease expression and progression rather than direct biological causality. Notably, anemia was associated with adverse kidney outcomes in men across the adult lifespan and in women aged ≥ 50 years. This pattern may reflect disease-specific characteristics of ADPKD as a genetic disorder, in addition to, rather than distinct from, general features of chronic kidney disease. Unlike many forms of CKD, in which the rate of kidney function decline often attenuates with aging, ADPKD is characterized by continuous structural and functional renal damage driven by lifelong cyst growth. In this context, anemia may serve as a cumulative marker of ongoing renal injury, highlighting the potential importance of sustained and individualized clinical assessment throughout the disease course.

Clinically, while current CKD guidelines recommend treating anemia at Hb levels < 10–11 g/dL [44–50], whether these thresholds are optimal for risk stratification in ADPKD remains unclear. Notably, lower hemoglobin cutoffs such as Hb < 11 g/dL may reduce prognostic discrimination in ADPKD by grouping patients with moderately reduced hemoglobin levels (e.g., 11–12 g/dL), who already carry increased risk, into the reference category. Our findings indicate that higher, sex-specific hemoglobin thresholds may enhance risk stratification, particularly in men, by better capturing clinically relevant heterogeneity, consistent with KDIGO’s 2024 emphasis on precision medicine approaches [22].

While our findings provide novel insights, several limitations should be acknowledged. First, the observational nature of this study precludes definitive causal inference. Second, the analyses were based on baseline data, and longitudinal changes in hemoglobin levels, kidney function, or treatment strategies were not evaluated. Third, although the cohort was relatively large for an ADPKD-specific study, it was derived from a single center, which may limit generalizability. However, this real-world single-center design may also capture clinically relevant heterogeneity, particularly in early-stage, pre-dialysis ADPKD populations. Fourth, because the primary analyses relied on sex and age-based cross-classification, additional metabolic comorbidities such as diabetes and dyslipidemia were not included in the main multivariable models to avoid overfitting in smaller subgroups; however, these factors were evaluated in sensitivity analyses in the overall cohort. Finally, the consistency of results across multiple sensitivity analyses—including those incorporating height-adjusted TKV and Mayo imaging classification—suggests that the observed sex- and age-specific prognostic impact of anemia is unlikely to be solely driven by baseline differences in kidney volume, particularly among younger men.

In conclusion, this study identified anemia as a significant prognostic marker for adverse renal outcomes in ADPKD. Although anemia has traditionally been viewed as a consequence of progressive kidney dysfunction, our findings—together with plausible pathophysiological considerations—indicate that lower hemoglobin levels are closely associated with disease progression in this population. Recognition of anemia, particularly in men, may therefore contribute to refined risk stratification and longitudinal monitoring in patients with ADPKD.

Given the lifelong and progressive nature of ADPKD, these findings underscore the importance of sustained and individualized assessment across the disease course. The observed sex-specific differences highlight the relevance of attribute-based approaches in understanding prognostic heterogeneity in ADPKD. Although anemia should be interpreted as a prognostic marker rather than a causal determinant of disease progression, our results provide a conceptual basis for future interventional studies to evaluate whether individualized hemoglobin management strategies may improve renal outcomes in ADPKD. Future studies are warranted to clarify the causal pathways linking anemia and kidney function decline and to determine whether targeted interventions may influence long-term renal outcomes.

## Supplementary Information

Below is the link to the electronic supplementary material.

Supplementary file1 (DOCX 1172 KB)
