# Supplementary material for: Attribute-based cross-classification reveals sex- and age-specific prognostic impact of anemia in ADPKD
Source: Clin Exp Nephrol. 2026 Apr 29;30(7):1091–100. doi: 10.1007/s10157-026-02867-0 (PMC13290795; doi:10.1007/s10157-026-02867-0)
Supplement: Supplementary file 1 — Supplementary file1 (DOCX 1172 KB) [file 10157_2026_2867_MOESM1_ESM.docx]

**Supplementary Material**

**Supplementary Material to “Attribute-Based Cross-Classification Reveals Sex- and Age-Specific Prognostic Impact of Anemia in ADPKD”**

Kosaku Nitta^1^, Hiroshi Kataoka^1,2^*, Yusuke Ushio (co-first author)^1^, Shun Manabe^1^*, Shiho Makabe^1^, Shigeru Otsubo^2^, Norio Hanafusa^3^, Ken Tsuchiya^3^, Junichi Hoshino^1^, Toshio Mochizuki^1,4^

^1^ Department of Nephrology, Tokyo Women’s Medical University, Tokyo, Japan

^2^ Department of Clinical Engineering, Faculty of Human Care at Makuhari, Tohto University, Chiba, Japan

^3^ Department of Blood Purification, Tokyo Women’s Medical University, Tokyo, Japan

^4^ PKD Nephrology Clinic, Tokyo, Japan

Hiroshi Kataoka, MD, PhD

Department of Nephrology, Tokyo Women’s Medical University, 8-1 Kawada-cho, Shinjuku-ku, Tokyo 162-8666, Japan

Tel.: +81-3-3353-8111, Fax: +81-3-3356-0293, E-mail: [kataoka@twmu.ac.jp](mailto:kataoka@twmu.ac.jp)

Shun Manabe, MD, PhD

Department of Nephrology, Tokyo Women’s Medical University, 8-1 Kawada-cho, Shinjuku-ku, Tokyo 162-8666, Japan

Tel.: +81-3-3353-8111, Fax: +81-3-3356-0293, E-mail: [shunmn5711@gmail.com](mailto:shunmn5711@gmail.com)

These Supplementary materials provide further methodological detail and results for the main paper.

**Supplementary Methods**

- Covariate assessments
- Assessment of total kidney volume
- Definition of comorbidities
- Statistical analyses

**Supplementary Figure 1.** Attribute-based cross-classification approach (Importance Grid Analysis).

**Supplementary Figure 2.** Patient selection flow chart.

**Supplementary Figure 3.** Kaplan–Meier curves for kidney outcomes stratified by hemoglobin status and cross-classified by sex and age in patients with ADPKD

**Supplementary Table 1.** Multivariable Cox regression analyses for kidney outcomes in patients aged ≥18 years (Sensitivity analysis)

**Supplementary Table 2.** Multivariable Cox regression analyses for kidney outcomes excluding patients treated with tolvaptan at baseline (Sensitivity analysis)

**Supplementary Table 3.** Multivariable Cox regression analyses for kidney outcomes using height-adjusted total kidney volume (htTKV) instead of TKV (Sensitivity analysis)

**Supplementary Table 4.** Multivariable Cox regression analyses for kidney outcomes using Mayo imaging classification (Classes 1C–1E) instead of TKV (Sensitivity analysis)

**Supplementary Table 5.** Multivariable Cox regression analyses for kidney outcomes including metabolic comorbidities (Sensitivity analysis)

**Supplementary Table 6.** Multivariable Cox regression analyses for kidney outcomes in attribute-based cross-classified sub-cohorts by sex and age among patients aged ≥18 years (Sensitivity analysis)

**Supplementary Method**

**Covariate Assessments**

During a regular outpatient clinic visit, anthropometric and physical examinations were conducted including assessments of blood pressure components and measurement of height and weight. All biochemical analyses were performed on samples obtained from patients after fasting overnight. The estimated glomerular filtration rate for Japanese patients was determined using the reference formula.^1^ Ttotal kidney volume, and definitions of comorbidities are presented as follows.

**Assessment of total kidney volume**

Total kidney volume (TKV) was estimated from the maximum length, width, and depth of the kidney, using the formula for an ellipsoid: π/6 × length × width × depth.

**Definition of comorbidities**

The impact of concomitant treatment and comorbidities was assessed ^2^. The comorbidities were recorded as positive according to the following criteria. Hypertension was defined as systolic BP ≥ 140 mmHg or diastolic BP ≥ 90 mmHg, or currently taking an antihypertensive agent; hyperuricemia was defined as serum uric acid level ≥7.0 mg/dL, or currently taking an anti-hypouricemic agent; hypertriglyceridemia was defined as serum TG level ≥ 150 mg/dL or currently taking an anti-dyslipidemic agent; low HDL-C was defined as a serum HDL-C level ≤ 40 mg/dL, or currently taking an anti-dyslipidemic agent; high LDL-C was defined as serum LDL-C level ≥ 140 mg/dL, or currently taking an anti-dyslipidemic agent; diabetes mellitus was defined as glycated hemoglobin level ≥6.5%, diagnosis of diabetes mellitus, or intake of an antidiabetic agent..

**Statistical analyses**

Competing risk of death was considered in the interpretation of the results. Because the number of deaths during follow-up was small (n=17), and Fine–Gray competing risk models were not available in the statistical software used (JMP Pro), analyses were performed with censoring at death. Given the low event rate for death, the impact of competing risk was considered unlikely to materially alter the observed associations.

**Supplementary References**

1. Matsuo, S. *et al.* Revised equations for estimated GFR from serum creatinine in Japan. *American journal of kidney diseases : the official journal of the National Kidney Foundation*. **53**, 982-992 (2009).

2. Ording, A. G. & Sorensen, H. T. Concepts of comorbidities, multiple morbidities, complications, and their clinical epidemiologic analogs. *Clinical epidemiology*. **5**, 199-203 (2013).


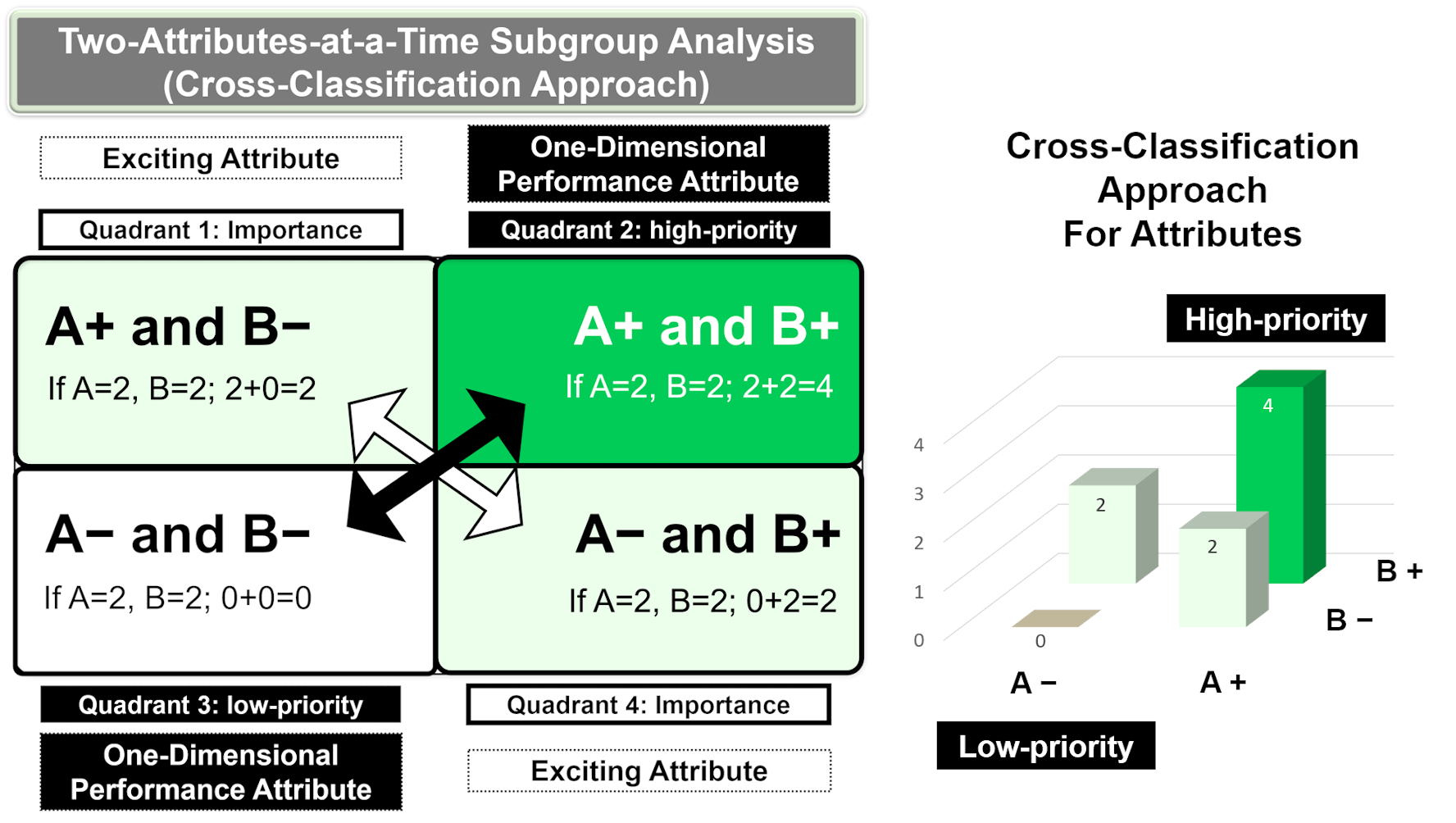


**Supplementary Figure 1. Attribute-based cross-classification approach (Importance Grid Analysis).**

Attributes positioned on the diagonal represent the most opposite characteristics. If attributes A and B are deemed important, an attribute containing both A and B will be considered the most important, while an attribute lacking both A and B will be regarded as the least important. For instance, if values are assigned to attributes A and B, with both set to 2, the bottom left section will have a value of 0 and the upper right section will have a value of 4.


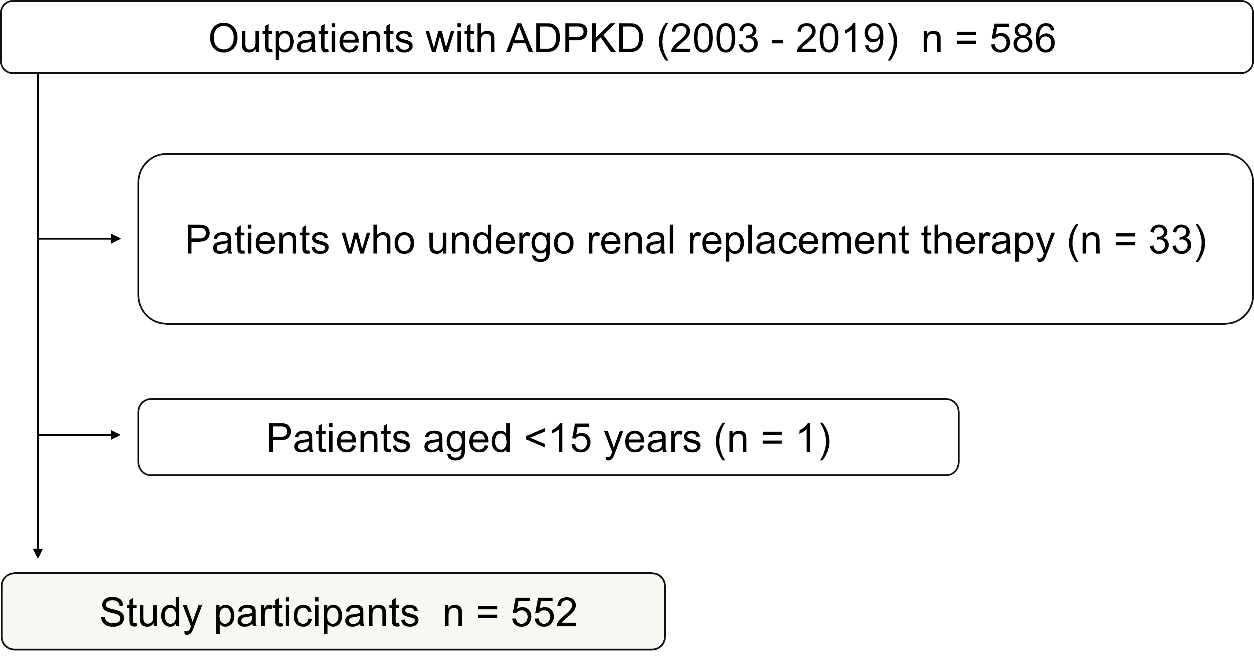


**Supplementary Figure 2. Patient selection flow chart.**

Abbreviations: ADPKD, autosomal dominant polycystic kidney disease


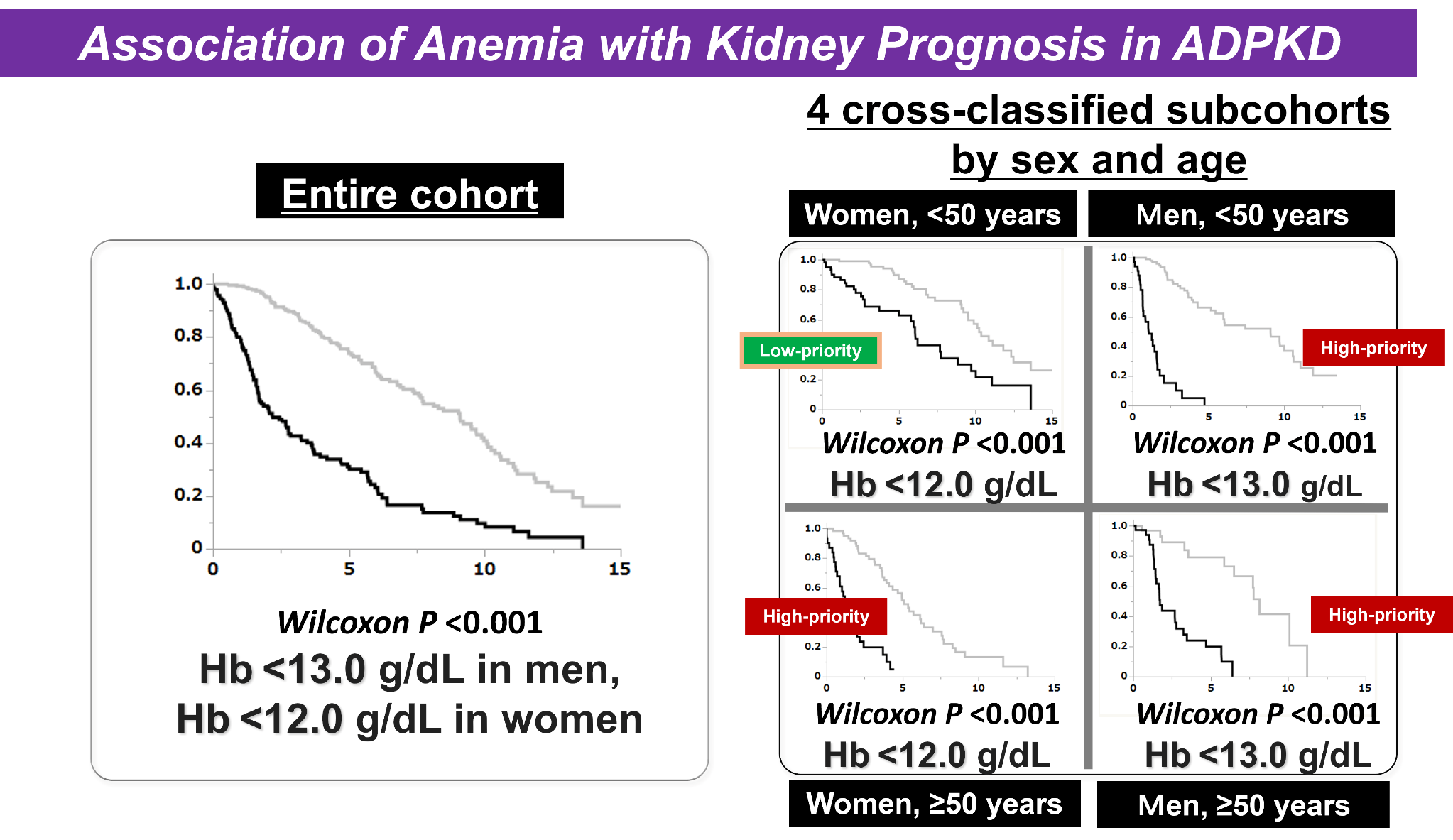


**Supplementary Figure 3.** Kaplan–Meier curves for kidney outcomes stratified by hemoglobin status and cross-classified by sex and age in patients with ADPKD

Kaplan–Meier curves illustrating kidney outcomes in patients with autosomal dominant polycystic kidney disease (ADPKD). Kaplan–Meier curves are presented for illustrative purposes to facilitate clinical interpretation of the multivariable findings. Hemoglobin thresholds were selected to enhance visualization of risk gradients and were not used to define the primary conclusions.The left panel shows results for the entire cohort, and the right panels show results for sub-cohorts cross-classified by sex and age (<50 and ≥50 years). Patients were stratified by anemia status using the hemoglobin cutoff values that yielded the highest hazard ratios in the corresponding multivariable analyses (Tables 2 and 3). Kidney outcome was defined as a ≥30% decline in estimated glomerular filtration rate or initiation of renal replacement therapy.

**Supplementary Table 1.** Multivariable Cox regression analyses for kidney outcomes in patients aged ≥18 years (Sensitivity analysis)

| **Variables** | **Hazard Ratio**  **(95% CI)** | ***P*–value** |
| --- | --- | --- |
| Age (10-year increase) | 0.80 (0.70–0.91) | 0.001* |
| Woman (vs. man) | 1.07 (0.73–1.58) | 0.727 |
| eGFR (10-mL/min/1.73m^2^ increase) | 0.64 (0.57–0.71) | <0.001* |
| U-Prot (grade 0–3) | 1.90 (1.52–2.37) | <0.001* |
| TKV (100 mL/m increase) | 1.02 (1.01–1.03) | 0.001* |
| Hypertension (vs. no) | 1.54 (1.12–2.12) | 0.008* |
| Uric acid (1 mg/dL increase) | 1.00 (0.88–1.14) | 0.940 |
| Hb (1 g/dL increase)： primary exposure | 0.83 (0.76–0.92) | <0.001* |
| Sensitivity analyses　 (categorical definitions of anemia; separate models) |  |  |
| Anemia of Hb <11.0 g/ dL | [1.38 (0.95–2.01)] | [0.094] |
| Anemia of Hb <12.0 g/ dL | [1.68 (1.18–2.39)] | [0.004*] |
| Anemia of Hb <13.0 g/ dL | [1.55 (1.08–2.24)] | [0.019*] |
| Anemia of Hb <12.0 g/dL in men, Hb <11.0 g/dL in women | [1.50 (1.04–2.16)] | [0.030*] |
| Anemia of Hb <13.0 g/dL in men, Hb <12.0 g/dL in women | [1.76 (1.24–2.48)] | [0.001*] |

**P* < 0.05. Analyses were performed in the analytic cohort with complete data for all covariates. The primary multivariable model analyzed hemoglobin as a continuous variable (follow-up duration = 5.7 years; n = 438, events = 218). Categorical anemia definitions were evaluated in separate multivariable models replacing continuous hemoglobin and are presented as sensitivity analyses. Variables representing established risk factors for kidney disease progression in autosomal dominant polycystic kidney disease and chronic kidney disease were included in the multivariable models. Hazard ratios shown in brackets [ ] represent results of prespecified sensitivity analyses using alternative hemoglobin thresholds (<11.0, <12.0, and <13.0 g/dL; <12.0 g/dL in men and <11.0 g/dL in women; and <13.0 g/dL in men and <12.0 g/dL in women) and were not included in the primary model. Anemia was defined by hemoglobin thresholds or the use of iron supplementation or erythropoiesis-stimulating agents. Abbreviations: CI, confidence interval; vs., versus eGFR, estimated glomerular filtration rate; Hb, hemoglobin; TKV, total kidney volume; U-Prot, urinary protein excretion.

**Supplementary Table 2.** Multivariable Cox regression analyses for kidney outcomes excluding patients treated with tolvaptan at baseline (Sensitivity analysis)

| **Variables** | **Hazard Ratio**  **(95% CI)** | ***P*–value** |
| --- | --- | --- |
| Age (10-year increase) | 0.80 (0.70–0.92) | 0.001* |
| Woman (vs. man) | 1.05 (0.71–1.55) | 0.802 |
| eGFR (10-mL/min/1.73m^2^ increase) | 0.64 (0.57–0.71) | <0.001* |
| U-Prot (grade 0–3) | 1.86 (1.48–2.32) | <0.001* |
| TKV (100 mL/m increase) | 1.02 (1.01–1.03) | 0.002* |
| Hypertension (vs. no) | 1.57 (1.14–2.16) | 0.006* |
| Uric acid (1 mg/dL increase) | 1.01 (0.89–1.15) | 0.837 |
| Hb (1 g/dL increase)： primary exposure | 0.83 (0.75–0.91) | <0.001* |
| Sensitivity analyses　 (categorical definitions of anemia; separate models) |  |  |
| Anemia of Hb <11.0 g/ dL | [1.36 (0.92–1.97)] | [0.118] |
| Anemia of Hb <12.0 g/ dL | [1.68 (1.18–2.39)] | [0.004*] |
| Anemia of Hb <13.0 g/ dL | [1.58 (1.09–2.28)] | [0.015*] |
| Anemia of Hb <12.0 g/dL in men, Hb <11.0 g/dL in women | [1.49 (1.02–2.15)] | [0.034*] |
| Anemia of Hb <13.0 g/dL in men, Hb <12.0 g/dL in women | [1.80 (1.27–2.54)] | [<0.001*] |

**P* < 0.05. Analyses were performed in the analytic cohort with complete data for all covariates. The primary multivariable model analyzed hemoglobin as a continuous variable (follow-up duration = 5.7 years; n = 438, events = 217). Categorical anemia definitions were evaluated in separate multivariable models replacing continuous hemoglobin and are presented as sensitivity analyses. Variables representing established risk factors for kidney disease progression in autosomal dominant polycystic kidney disease and chronic kidney disease were included in the multivariable models. Hazard ratios shown in brackets [ ] represent results of prespecified sensitivity analyses using alternative hemoglobin thresholds (<11.0, <12.0, and <13.0 g/dL; <12.0 g/dL in men and <11.0 g/dL in women; and <13.0 g/dL in men and <12.0 g/dL in women) and were not included in the primary model. Anemia was defined by hemoglobin thresholds or the use of iron supplementation or erythropoiesis-stimulating agents. Abbreviations: CI, confidence interval; ; vs., versus eGFR, estimated glomerular filtration rate; Hb, hemoglobin; TKV, total kidney volume; U-Prot, urinary protein excretion.

**Supplementary Table 3.** Multivariable Cox regression analyses for kidney outcomes using height-adjusted total kidney volume (htTKV) instead of TKV (Sensitivity analysis)

| **Variables** | **Hazard Ratio**  **(95% CI)** | ***P*–value** |
| --- | --- | --- |
| Age (10-year increase) | 0.80 (0.70–0.91) | 0.001* |
| Woman (vs. man) | 1.02 (0.71–1.55) | 0.902 |
| eGFR (10-mL/min/1.73m^2^ increase) | 0.64 (0.58–0.70) | <0.001* |
| U-Prot (grade 0–3) | 1.86 (1.50–2.33) | <0.001* |
| TKV (100 mL/m increase) | 1.04 (1.01–1.06) | <0.001* |
| Hypertension (vs. no) | 1.57 (1.14–2.17) | 0.006* |
| Uric acid (1 mg/dL increase) | 1.02 (0.89–1.16) | 0.784 |
| Hb (1 g/dL increase)： primary exposure | 0.82 (0.75–0.91) | 0.001* |
| Sensitivity analyses　 (categorical definitions of anemia; separate models) |  |  |
| Anemia of Hb <11.0 g/ dL | [1.41 (0.96–2.05)] | [0.072] |
| Anemia of Hb <12.0 g/ dL | [1.75 (1.23–2.49)] | [0.002*] |
| Anemia of Hb <13.0 g/ dL | [1.67 (1.15–2.42)] | [0.007*] |
| Anemia of Hb <12.0 g/dL in men, Hb <11.0 g/dL in women | [1.54 (1.06–2.22)] | [0.022*] |
| Anemia of Hb <13.0 g/dL in men, Hb <12.0 g/dL in women | [1.88 (1.33–2.66)] | [<0.001*] |

**P* < 0.05. Analyses were performed in the analytic cohort with complete data for all covariates. The primary multivariable model analyzed hemoglobin as a continuous variable (follow-up duration = 6.1 years; n = 430, events = 216). Categorical anemia definitions were evaluated in separate multivariable models replacing continuous hemoglobin and are presented as sensitivity analyses. Variables representing established risk factors for kidney disease progression in autosomal dominant polycystic kidney disease and chronic kidney disease were included in the multivariable models. Hazard ratios shown in brackets [ ] represent results of prespecified sensitivity analyses using alternative hemoglobin thresholds (<11.0, <12.0, and <13.0 g/dL; <12.0 g/dL in men and <11.0 g/dL in women; and <13.0 g/dL in men and <12.0 g/dL in women) and were not included in the primary model. Anemia was defined by hemoglobin thresholds or the use of iron supplementation or erythropoiesis-stimulating agents. Abbreviations: CI, confidence interval; ; vs., versus eGFR, estimated glomerular filtration rate; Hb, hemoglobin; htTKV, height-adjusted total kidney volume; U-Prot, urinary protein excretion.

**Supplementary Table 4.** Multivariable Cox regression analyses for kidney outcomes using Mayo imaging classification (Classes 1C–1E) instead of TKV (Sensitivity analysis)

| **Variables** | **Hazard Ratio**  **(95% CI)** | ***P*–value** |
| --- | --- | --- |
| Age (10-year increase) | 0.92 (0.80–1.08) | 0.345 |
| Woman (vs. man) | 0.81 (0.55–1.22) | 0.315 |
| eGFR (10-mL/min/1.73m^2^ increase) | 0.65 (0.58–0.72) | <0.001* |
| U-Prot (grade 0–3) | 1.89 (1.51–2.35) | <0.001* |
| TKV (100 mL/m increase) | 1.81 (1.31–2.50) | <0.001* |
| Hypertension (vs. no) | 1.52 (1.10–2.10) | 0.011* |
| Uric acid (1 mg/dL increase) | 0.98 (0.71–0.87) | 0.725 |
| Hb (1 g/dL increase)： primary exposure | 0.79 (0.71–0.87) | <0.001* |
| Sensitivity analyses　 (categorical definitions of anemia; separate models) |  |  |
| Anemia of Hb <11.0 g/ dL | [1.53 (1.02–2.28)] | [0.038*] |
| Anemia of Hb <12.0 g/ dL | [2.02 (1.40–2.91)] | [<0.001*] |
| Anemia of Hb <13.0 g/ dL | [1.84 (1.26–2.70)] | [0.002*] |
| Anemia of Hb <12.0 g/dL in men, Hb <11.0 g/dL in women | [1.80 (1.23–2.64)] | [0.003*] |
| Anemia of Hb <13.0 g/dL in men, Hb <12.0 g/dL in women | [2.10 (1.47–3.00)] | [<0.001*] |

**P* < 0.05. Analyses were performed in the analytic cohort with complete data for all covariates. The primary multivariable model analyzed hemoglobin as a continuous variable (follow-up duration = 6.9 years; n = 425, events = 215). Categorical anemia definitions were evaluated in separate multivariable models replacing continuous hemoglobin and are presented as sensitivity analyses. Variables representing established risk factors for kidney disease progression in autosomal dominant polycystic kidney disease and chronic kidney disease were included in the multivariable models. Hazard ratios shown in brackets [ ] represent results of prespecified sensitivity analyses using alternative hemoglobin thresholds (<11.0, <12.0, and <13.0 g/dL; <12.0 g/dL in men and <11.0 g/dL in women; and <13.0 g/dL in men and <12.0 g/dL in women) and were not included in the primary model. Anemia was defined by hemoglobin thresholds or the use of iron supplementation or erythropoiesis-stimulating agents. Abbreviations: CI, confidence interval; ; vs., versus eGFR, estimated glomerular filtration rate; Hb, hemoglobin; U-Prot, urinary protein excretion.

**Supplementary Table 5.** Multivariable Cox regression analyses for kidney outcomes including metabolic comorbidities (Sensitivity analysis)

| **Variables** | **Hazard Ratio**  **(95% CI)** | ***P*–value** |
| --- | --- | --- |
| Age (10-year increase) | 0.78 (0.68–0.89) | <0.001* |
| Woman (vs. man) | 1.11 (0.75–1.66) | 0.591 |
| eGFR (10-mL/min/1.73m^2^ increase) | 0.63 (0.56–0.70) | <0.001* |
| U-Prot (grade 0–3) | 1.84 (1.46–2.32) | <0.001* |
| TKV (100 mL/m increase) | 1.02 (1.00–1.03) | 0.011* |
| Hypertension (vs. no) | 1.59 (1.15–2.21) | 0.005* |
| Uric acid (1 mg/dL increase) | 0.99 (0.87–1.14) | 0.938 |
| Body mass index (1 kg/m^2^ increase) | 1.03 (0.98–1.09) | 0.185 |
| Hypertriglyceridemia (vs. no) | 1.05 (0.73–1.52) | 0.776 |
| Low HDL cholesterol (vs. no) | 1.01 (0.64–1.59) | 0.725 |
| High LDL cholesterol (vs. no) | 0.98 (0.71–0.87) | 0.970 |
| Diabetes (vs. no) | 0.40 (0.19–0.85) | 0.017* |
| Hb (1 g/dL increase)： primary exposure | 0.81 (0.73–0.89) | <0.001* |
| Categorical analyses (alternative hemoglobin definitions; separate models) |  |  |
| Anemia of Hb <11.0 g/ dL | [1.42 (0.97–2.07)] | [0.073] |
| Anemia of Hb <12.0 g/ dL | [1.96 (1.36–2.81)] | [<0.001*] |
| Anemia of Hb <13.0 g/ dL | [1.69 (1.15–2.48)] | [0.007*] |
| Anemia of Hb <12.0 g/dL in men, Hb <11.0 g/dL in women | [1.58 (1.08–2.29)] | [0.017*] |
| Anemia of Hb <13.0 g/dL in men, Hb <12.0 g/dL in women | [2.05 (1.43–2.91)] | [<0.001*] |

**P* < 0.05. Analyses were performed in the analytic cohort with complete data for all covariates. The primary multivariable model analyzed hemoglobin as a continuous variable (follow-up duration = 5.7 years; n = 429, events = 216). Categorical anemia definitions were evaluated in separate multivariable models replacing continuous hemoglobin and are presented as secondary analyses. Variables representing established risk factors for kidney disease progression in autosomal dominant polycystic kidney disease and chronic kidney disease were included in the multivariable models. Hazard ratios shown in brackets [ ] represent results of prespecified sensitivity analyses using alternative hemoglobin thresholds (<11.0, <12.0, and <13.0 g/dL; <12.0 g/dL in men and <11.0 g/dL in women; and <13.0 g/dL in men and <12.0 g/dL in women) and were not included in the primary model. Anemia was defined by hemoglobin thresholds or the use of iron supplementation or erythropoiesis-stimulating agents. Abbreviations: CI, confidence interval; ; vs., versus eGFR, estimated glomerular filtration rate; Hb, hemoglobin; U-Prot, urinary protein excretion.

**Supplementary Table 6.** Multivariable Cox regression analyses for kidney outcomes in attribute-based cross-classified sub-cohorts by sex and age among patients aged ≥18 years (Sensitivity analysis)

|  | **Men, <50 years**  **follow-up = 5.3 years**  **n=143, event=66** | | **Men, ≥50 years**  **follow-up = 3.9 years**  **n=61, event=36** | | **Women, <50 years**  **follow-up = 6.5 years**  **n=165, event=65** | | **Women, ≥50 years**  **follow-up = 4.8 years**  **n=82, event=56** | |
| --- | --- | --- | --- | --- | --- | --- | --- | --- |
| **Variables** | Hazard Ratio  (95% CI) | *P*–  value | Hazard Ratio  (95% CI) | *P*–  value | Hazard Ratio  (95% CI) | *P*–  value | Hazard Ratio  (95% CI) | *P*–  value |
| Age (10-year increase) | 0.68 (0.43–1.08) | 0.097 | 0.78 (0.51–1.16) | 0.228 | 1.00 (0.64–1.59) | 0.996 | 0.73 (0.48–1.10) | 0.134 |
| eGFR (10-mL/min/1.73m^2^ increase) | 0.59 (0.46–0.75) | <0.001* | 0.69 (0.44–1.01) | 0.076 | 0.74 (0.60–0.91) | 0.004* | 0.61 (0.48–0.77) | <0.001* |
| U-Prot (grade 0–3) | 1.41 (0.96–2.04) | 0.072 | 2.64 (1.43–4.81) | 0.002* | 1.35 (0.75–2.36) | 0.306 | 3.55 (2.24–5.60) | <0.001* |
| TKV (100 mL/m increase) | 1.02 (1.00–1.04) | 0.014* | 0.98 (0.94–1.02) | 0.333 | 1.10 (1.05–1.15) | <0.001* | 0.99 (0.95–1.03) | 0.644 |
| Hypertension (vs. no) | 2.48 (1.30–5.05) | 0.008* | 0.52 (0.22–1.32) | 0.149 | 1.04 (0.55–1.90) | 0.907 | 1.50 (0.79–2.91) | 0.220 |
| Uric acid (1 mg/dL increase) | 1.03 (0.85–1.24) | 0.751 | 0.88 (0.65–1.20) | 0.396 | 0.95 (0.70–1.30) | 0.753 | 1.23 (0.91–1.67) | 0.187 |
| Hb (1 g/dL increase) | [0.78 (0.63–0.95)] | [0.015*] | [0.77 (0.54–1.12)] | [0.163] | [0.86 (0.69–1.06)] | [0.150] | [0.69 (0.52–0.91)] | [0.008*] |
| Anemia of Hb <11.0 g/ dL | [1.83 (0.69–4.78)] | [0.222] | [1.24 (0.40–3.52)] | [0.696] | [1.16 (0.55–2.43)] | [0.703] | [2.17 (0.90–5.09)] | [0.078] |
| Anemia of Hb <12.0 g/ dL | [1.65 (0.60–4.68)] | [0.336] | [2.45 (0.93–6.57)] | [0.069] | **1.98** (1.04–3.68) ： primary exposure | 0.035* | **2.08** (1.01–4.29) ： primary exposure | 0.045* |
| Anemia of Hb <13.0 g/ dL | **2.92** (1.13–7.61) ： primary exposure | 0.028* | **3.84** (1.29–12.03) ： primary exposure | 0.017* | [1.15 (0.62–2.18)] | [0.657] | [0.98 (0.40–2.54)] | [0.969] |

**P* < 0.05. Analyses were conducted in patients aged ≥18 years. All analyses were performed in the analytic cohort with complete data for all covariates. For hemoglobin, three prespecified, clinically relevant thresholds (<11.0, <12.0, and <13.0 g/dL) were evaluated in each sex- and age-stratified subgroup. The multivariable analyses present the results for the threshold associated with the highest hazard ratio within this prespecified range, which is shown as the primary result. Hemoglobin analyzed as a continuous variable and the remaining thresholds are shown in brackets [ ] as sensitivity analyses. Variables representing established risk factors for kidney disease progression in autosomal dominant polycystic kidney disease and chronic kidney disease were included in all models. Anemia was defined by hemoglobin thresholds and/or the use of iron supplementation or erythropoiesis-stimulating agents. Abbreviations: CI, confidence interval; eGFR, estimated glomerular filtration rate; Hb, hemoglobin; U-Prot, urinary protein excretion; vs., versus.
